# Variants in the APOB gene was associated with Ischemic Stroke susceptibility in Chinese Han male population

**DOI:** 10.18632/oncotarget.23369

**Published:** 2017-12-18

**Authors:** Feng Zhou, Tie Guo, Lv Zhou, Yanhui Zhou, Dan Yu

**Affiliations:** ^1^ Department of Neurology, Affiliated Haikou Hospital at Xiangya Medical College, Central South University, Haikou 570208, Hainan, China

**Keywords:** Ischemic Stroke, APOB, gene polymorphism, massARRAY

## Abstract

**Background:**

Stroke is an extremely complicated disease caused by multiple factors. Epidemiological studies have shown that genetic factors contribute to the pathogenesis of stroke. There is still little research on the effect of *ApoB* gene on stroke in Chinese Han population. The purpose of our research was to explore the effect of *ApoB* gene polymorphism on the genetic susceptibility to Ischemic Stroke in Chinese Han male population.

**Materials and methods:**

7 polymorphisms in *ApoB* gene were selected and genotyped using Sequenom MassARRAY in 325 ischemic stroke male patients and 399 healthy male controls in Chinese Han population. The association between *ApoB* gene and genetic susceptibility to Ischemic Stroke was performed by the χ^2^ test, genetic model analysis and haplotype analysis.

**Results:**

In the allele model, *ApoB* rs1042034 “T” allele and rs673548 “G” allele increased the risk of the Ischemic Stroke (rs1042034: OR=1.29, 95%CI: 1.02-1.63, p=0.030; rs673548: OR=1.28, 95%CI: 1.02-1.62, p=0.034). Logistic regression analysis found that *ApoB* rs1042034 and rs673548 increased the risk of Ischemic Stroke in the log-additive model, the odds of having Ischemic Stroke would be 1.28-fold and 1.27-fold with the variant allele, respectively. We also found that the risk of individuals carrying the *ApoB* rs693 “AA-AG” genotype had Ischemic Stroke risk of 1.52-fold of carrying “GG” genotype in the dominant model. The haplotype analysis shown that “TAG” haplotype raised the risk of Ischemic Stroke (OR=1.52, 95%CI: 1.02-2.27, p=0.0042).

**Conclusion:**

The polymorphisms of the *ApoB* gene may affect Ischemic Stroke occurrence.

## INTRODUCTION

Strokes are one of the three most common causes of death and the major cause of adult chronic disability. A stroke is a sudden onset of cerebral blood circulation disruption. Once a stroke has occurred, most affected individuals suffer from disability, cognitive dysfunction and other complications and have a higher risk of death. About 15 million people suffer from strokes for the first time each year, of which 1/3, or about 6.6 million, result in death [1]. Risk factors for coronary artery disease (CAD) and ischemic stroke include hypertension, diabetes, dyslipidemia, and metabolic syndrome [2]. Dyslipidemia, i.e., elevated levels of total cholesterol, triglycerides, low-density lipoprotein and lipoprotein, and a decrease in HDL levels are risk factors for stroke [3, 4]. At present, more and more attention has been paid to the genetic factors of Ischemic Stroke.

The *ApoB* gene is located on the short arm of human chromosome 2, with a length of 43kb. The expression products of ApoB gene are mainly divided into Apo B100 and Apo B48. Apo B100 is a regulatory factor involved in thrombosis. Apo B48 participates in the synthesis, assembly and secretion of VLDL and participates in the digestion, absorption and transportation of exogenous lipids. One research consisted of 1128 people, and revealed that *ApoB* rs1042034 loci was significant associated with plasma cholesterol [5]. Another study found that oxLDL influence the incidence of stroke [6], and rs676210 in *ApoB* gene associated with oxLDL [7], so rs676210 loci may affect the incidence risk of stroke. Two small case-control studies [8, 9] and a small cohort study of patients with Ischemic stroke found that elevated apoB was associated with an increased risk Ischemic stroke [10].

However, there is little research on the relationship between *ApoB* gene and ischemic stroke in Chinese people. So, the purpose of our research was to explore the effect of *ApoB* gene polymorphism on the genetic susceptibility to Ischemic Stroke in Chinese Han male population.

## RESULTS

Table 1 shown that 7 SNPs were met HWE. In the allele model, *ApoB* rs1042034 “T” allele and rs673548 “G” allele increased the risk of the Ischemic Stroke (rs1042034: OR=1.29, 95%CI: 1.02-1.63, *p*=0.030; rs673548: OR=1.28, 95%CI: 1.02-1.62, *p*=0.034).

**Table 1:** Basic SNPs in APOB gene summary of all study participants

SNP rs#	Chr	Position	Band	Alleles A/B	Role	Amino acid change	Amino acid position	H-W *p*	OR(95%CI)	*p*
rs1042034	2	21225281	2p24.1	T/C	Coding exon	S/?	4338	0.3519	1.29(1.02-1.63)	0.03
rs1801702	2	21225485	2p24.1	G/C	Coding exon	R/T	4270	1	0.92(0.47-1.81)	0.807
rs693	2	21232195	2p24.1	A/G	Coding exon	T/?	2515	0.6686	1.44(0.97-2.12)	0.069
rs673548	2	21237544	2p24.1	G/A	Intron (boundary)	--	--	0.3531	1.28(1.02-1.62)	0.034
rs3791981	2	21245367	2p24.1	G/A	Intron	--	--	1	1.09(0.66-1.8)	0.744
rs679899	2	21250914	2p24.1	G/A	Coding exon	A/?	618	0.8514	1.22(0.93-1.6)	0.155
rs512535	2	21267782	2p24.1	T/C	Promoter	--	--	0.6662	1.18(0.92-1.5)	0.192

A/B stands for minor/major alleles on the control sample frequencies.

SNPs are excluded at 5% HWE *P* level.

*p* < 0.05 indicates statistical significance.

Through the genetic models analyses as shown Table 2, we found that *ApoB* rs1042034 and rs673548 increased the risk of Ischemic Stroke under the log-additive model, the odds of having Ischemic Stroke would be 1.28-fold (OR=1.28, 95%CI: 1.02-1.60, *p*=0.034) and 1.27-fold (OR=1.27, 95%CI: 1.01-1.60, *p*=0.038) with the variant allele, respectively. Meanwhile, we also found that the risk of individuals carrying the *ApoB* rs693 “AA-AG” genotype had Ischemic Stroke risk of 1.52-fold of carrying GG genotype (OR=1.52, 95%CI: 1.00-2.30, *p*=0.047) under the dominant model.

**Table 2:** Association of APOB SNPs with Ischemic Stroke risk based on logistical tests

SNP	Model	Genotype	Control	Case	Crude analysis		Adjusted analysis	
					OR (95% CI)	*p*-value	OR (95% CI)	*p*-value
rs1042034	Codominant	C/C	227 (56.9%)	161 (49.5%)	1	0.11	1	0.2
		T/C	143 (35.8%)	131 (40.3%)	1.29 (0.95-1.76)		1.42 (0.96-2.10)	
		T/T	29 (7.3%)	33 (10.2%)	1.60 (0.94-2.75)		1.27 (0.64-2.50)	
	Dominant	C/C	227 (56.9%)	161 (49.5%)	1	**0.048**	1	0.078
		T/C-T/T	172 (43.1%)	164 (50.5%)	1.34 (1.00-1.80)		1.39 (0.96-2.01)	
	Recessive	C/C-T/C	370 (92.7%)	292 (89.8%)	1	0.17	1	0.79
		T/T	29 (7.3%)	33 (10.2%)	1.44 (0.86-2.43)		1.09 (0.56-2.12)	
	Log-additive	---	---	---	1.28 (1.02-1.60)	**0.034**	1.24 (0.93-1.65)	0.14
rs693	Codominant	G/G	350 (87.7%)	268 (82.5%)	1	0.11	1	0.16
		G/A	47 (11.8%)	56 (17.2%)	1.56 (1.02-2.37)		1.65 (0.98-2.79)	
		A/A	2 (0.5%)	1 (0.3%)	0.65 (0.06-7.24)		0.75 (0.04-13.23)	
	Dominant	G/G	350 (87.7%)	268 (82.5%)	1	**0.047**	1	0.068
		G/A-A/A	49 (12.3%)	57 (17.5%)	1.52 (1.00-2.30)		1.62 (0.96-2.71)	
	Recessive	G/G-G/A	397 (99.5%)	324 (99.7%)	1	0.68	1	0.8
		A/A	2 (0.5%)	1 (0.3%)	0.61 (0.06-6.78)		0.69 (0.04-12.27)	
	Log-additive	---	---	---	1.45 (0.97-2.15)	0.067	1.53 (0.94-2.50)	0.09
rs673548	Codominant	A/A	225 (56.7%)	159 (49.2%)	1	0.11	1	0.22
		A/G	143 (36%)	132 (40.9%)	1.31 (0.96-1.78)		1.41 (0.96-2.09)	
		G/G	29 (7.3%)	32 (9.9%)	1.56 (0.91-2.68)		1.23 (0.62-2.45)	
	Dominant	A/A	225 (56.7%)	159 (49.2%)	1	**0.046**	1	0.088
		A/G-G/G	172 (43.3%)	164 (50.8%)	1.35 (1.00-1.81)		1.38 (0.95-1.99)	
	Recessive	A/A-A/G	368 (92.7%)	291 (90.1%)	1	0.21	1	0.85
		G/G	29 (7.3%)	32 (9.9%)	1.40 (0.83-2.36)		1.07 (0.55-2.08)	
	Log-additive	---	---	---	1.27 (1.01-1.60)	**0.038**	1.23 (0.92-1.63)	0.16

*p* < 0.05 indicates statistical significance.

**Figure 1:**
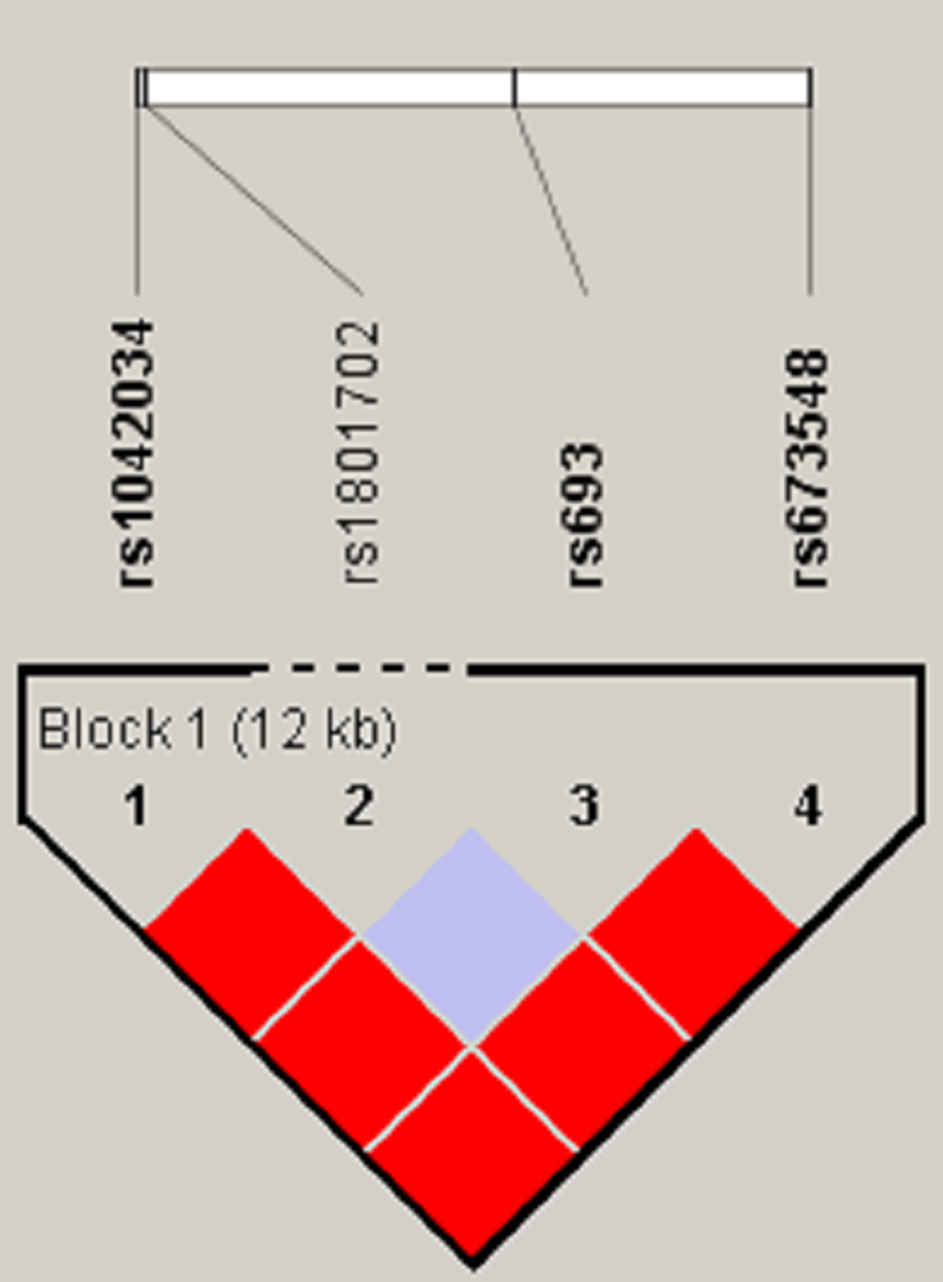
Haplotype block for the SNPs of ApoB

The linkage disequilibrium degree between two SNP loci was measured by the parameter D’ and R^2^, and the haplotype block was divided by D’ confidence interval method. Figure 1 shown that rs1042034, rs693, and rs673548 are highly linked, and further haplotype analysis revealed that haplotype “TAG” increased the risk of Ischemic stroke compared the haplotype “CGA” (OR=1.52, 95%CI: 1.02-2.27, *p*=0.0042) (Table 3).

**Table 3:** ApoB haplotype frequencies and their association with Ischemic Stroke

	Haplotype	Freq	Crude analysis		Adjusted analysis	
			OR (95% CI)	*p*-value	OR (95% CI)	*p*-value
rs1042034/rs693/rs673548	CGA	0.7238	1		1	
	TGG	0.1989	1.20 (0.93 - 1.55)	0.16	1.13 (0.82 - 1.56)	0.46
	TAG	0.0753	1.52 (1.02 - 2.27)	0.042	1.57 (0.96 - 2.58)	0.072

*p* < 0.05 indicates statistical significance.

## DISCUSSION

Some studies found that elevated apoB level added the risk of Ischemic stroke [8–10]. We investigated the effects of ApoB gene on susceptibility to Ischemic stroke in Chinese male population, and found that rs1042034, rs693 and rs673548 in *ApoB* gene increased the risk of Ischemic stroke in Chinese Han population.

The AP69 gene rs693 loci (C> T, synonymous mutation) is located in exon 26 of the ApoB gene, closely related to blood lipid level. Kathiresan and his colleagues found that the levels of LDL-C and TG of the rs693 T allele carriers were higher than the rs693 C allele carriers [11]. Rodrigues et al. found that rs693 locus T allele carriers had higher levels of TC and LDL-C by case-control analysis [12]. Boekholdt et al. [13] found that Homozygotes for the XbaI elevated levels of LDL cholesterol (LDL-C) and apoB, but decreased the risk of ischemic heart disease. In our data, rs673548 also showed significant associations with Ischemic Stroke risk, and rs693 and rs673548 have stronger linkage.

We also found that *ApoB* rs1042034, a missense resulting in Ser4338Asn, increased the risk of Ischemic stroke, the odds of having Ischemic Stroke would be 1.28-fold with the variant allele. In the study of Kim, which including 1128 participants, revealed that *ApoB* rs1042034 loci was significant associated with plasma cholesterol [5]. Kulminski AM et al. tracing the cardiovascular disease in the United States found that the rs1042034 “CC” genotype of ApoB gene is related to the increase of serum total cholesterol in young population, but the protective effect on lipid metabolism in elderly people [14]. So, we speculate that *ApoB* influences the risk of occurrence of Ischemic Stroke by influencing the concentration of lipoprotein.

In conclusion, our study revealed that *ApoB* rs1042034, rs693 and rs673548 mutation were significantly associations with the hereditary susceptibility of Ischemic stroke. Although sample size is small, our research has certain significance. In the future we will increase the sample size for validation studies.

## MATERIALS AND METHODS

### Study participants

A case-control study involving a Chinese study population of 325 ischemic stroke male patients (age mean: 63.44 year) and 399 healthy male controls (age mean: 47.57 year) was conducted at the Affiliated Haikou Hospital at Xiangya medical college, Central South University. All included patients were diagnosed according to the International Classification of Disease (9th revision, codes 430 to 438) on the basis of history, clinical symptoms, physical examination, and cranial computed tomography (CT) or magnetic resonance imaging (MRI). Patients with hemorrhagic stroke, subarachnoid hemorrhage, transient ischemic attack, traumatic brain injuries, infectious diseases, and tumors were excluded in this study. The controls were recruited from the Physical Examination Department in the same hospital. None of the controls showed evidence of stroke or other neurological diseases. Control participants with tumors, autoimmune diseases, liver ailments, nephrosis, or hematological diseases were excluded.

All of the participants signed an informed consent agreement. The Human Research Committee for Approval of Research Involving Human Subjects, Affiliated Haikou Hospital at Xiangya medical college, Central South University approved the use of human tissue in this study.

### SNP selection and genotyping

We selected seven candidate SNPs of *ApoB* according to previous published papers which demonstrated association with lipid metabolism abnormality related diseases and minor allele frequencies >5% in the HapMap Chinese Han Beijing population. We used the GoldMag-Mini Whole Blood Genomic DNA Purification Kit (GoldMag Co. Ltd. Xi’an City, China) extracted from whole blood. Using a NanoDrop 2000 (Gene Company Limited) were measured DNA concentrations. We used SequenomMassARRAY Assay Design 3.0 Software to design a Multiplexed SNP MassEXTEND assay [15]. SequenomMassARRAY RS1000 was used for genotyping, and the related data were managed using SequenomTyper 4.0 Software [15, 16]. Laboratory personnel were blinded to the genotyping results of all samples.

### Statistical analysis

Data analysis was performed using Microsoft Excel (Redmond, WA, USA) and SPSS 19.0 statistical package (SPSS, Chicago, IL, USA). All p values were two-sided, and p <0.05 was indicated statistical significance. Each SNP frequency in the control subjects was assessed for departure from Hardy–Weinberg Equilibrium (HWE) using an exact test. We calculated genotype frequencies of cases and controls using a χ^2^ test [17]. Odds ratios (ORs) and 95 % confidence intervals (CIs) were determined using unconditional logistic regression with adjustment for age and sex [18].

Five genetic models (genotype, dominant, recessive, and additive model) were performed using PLINK software (http://pngu.mgh.harvard.edu/purcell/plink/), to characterize the potential association of each *ApoB* polymorphism with the risk of esophageal carcinoma. Finally, we used Haploview software package (version 4.2) to evaluate pairwise linkage disequilibrium (LD), haplotype construction, and genetic association at the polymorphic loci [19, 20].
